# Supplementary material for: Content-rich biological network constructed by mining PubMed abstracts
Source: BMC Bioinformatics. 2004 Oct 8;5:147. doi: 10.1186/1471-2105-5-147 (PMC528731; doi:10.1186/1471-2105-5-147)
Supplement: Additional File 5 — The original Chilibot query results of the term "long-term potentiation (LTP)" and 22 other terms, limiting the latest references analyzed to the years 1990, 1995, 2000, and 2004. [file 1471-2105-5-147-S5.bz2 › chilibotAdditionalFile5/ltp2000/html/ACTIN_ACTININ.html]

 


 **ACTIN** and **ACTININ** 
  
Found 932 abstracts in PubMed,  **30 abstracts were retrieved and analyzed**.  


---

 Search Google  |
 PDF files only 
|  EDU domain only 

---

**Interactive relationship** (e.g. stimulation, inhibition, etc)

- We conclude that calponin and alpha  **actinin**  can coexist on  **actin** .  Ref: 10995029 IUBMB Life, 2000
- Immobilized alpha  **actinin**  can bind to  **actin**  filaments reversibly and ultimately stop the filaments from moving.  Ref: 10970781 Biochem J, 2000
- The results indicate that CLP36 binds via alpha  **actinin**  1 to  **actin**  filaments and stress fibers in activated human platelets and endothelial cells.  Ref: 11110697 Blood, 2000
- Simultaneous interaction of  **actin**  with alpha  **actinin**  and calponin.  Ref: 10995029 IUBMB Life, 2000
- Interaction of calponin and alpha  **actinin**  with  **actin**  was analyzed by means of cosedimentation and electron microscopy.  Ref: 10995029 IUBMB Life, 2000
- Binding of calponin to the single or bundled  **actin**  filaments was not dependent on the presence of alpha  **actinin**  and did not displace alpha  **actinin**  from  **actin** .  Ref: 10995029 IUBMB Life, 2000
- Finally, alpha  **actinin**  co localizes with  **actin**  and with PLD2 in the detergent insoluble fraction from sarcolemmal membranes.  Ref: 10801846 J Biol Chem, 2000
- In vitro cosedimentation studies also demonstrated that nebulin SH3 fragments did not bind to F alpha  **actin**  or alpha  **actinin** .  Ref: 10931867 J Cell Biol, 2000
- Pull down assays using the glutathione S transferase fusion system also showed the interaction of the PDZ domain of ENH with  **actin**  and alpha  **actinin** .  Ref: 10833443 Biochem Biophys Res Commun, 2000
- In muscle, titin acts as a molecular ruler organizing the  **actin**  cytoskeleton via interactions with many sarcomeric proteins, including the crosslinking protein alpha  **actinin** .  Ref: 11101506 EMBO J, 2000
- We identify a 30 residue Z repeat homologous sequence between the  **actin**  binding and rod regions of alpha  **actinin**  that binds the C terminal domain with nanomolar affinity.  Ref: 11101506 EMBO J, 2000
- **Actin**  binding was independent of the presence of tropomyosin and the  **actin**  cross linking proteins filamin and alpha  **actinin** .  Ref: 11096113 J Biol Chem, 2001
- The concentration of alpha  **actinin**  needed to stop all filaments from moving 0.8 microg ml with  **actin**  was very consistent both within and between experiments.  Ref: 10970781 Biochem J, 2000
- G  **actin**  was polymerized in the presence of calponin, alpha  **actinin** , or both of these  **actin**  binding proteins ABPs.  Ref: 10995029 IUBMB Life, 2000
- In the Z disk, it interacts with alpha  **actinin**  homodimers that are a principal component of the Z filaments linking  **actin**  filaments.  Ref: 10819994 Biochemistry, 2000
- Drebrin and alpha  **actinin**  2,  **actin**  binding proteins concentrated in spines, were also dependent on F  **actin**  for synaptic localization but were unaffected by detergent extraction.  Ref: 10844024 J Neurosci, 2000
- By immunoblotting and immunoprecipitation with specific antibodies we identified the presence of tropomyosin, myosin, a  **actinin** , and two different actins in the eluate corresponding to F  **actin**  binding proteins.  Ref: 10800190 Mem Inst Oswaldo CruzMem Inst Oswaldo Cruz, 2000
- Altogether, these results indicate that  **actinin**  4 and  **actinin**  1 contribute differently to F  **actin**  dynamics, that  **actinin**  4 is more preferentially involved in early stages of macropinocytosis than  **actinin**  1.  Ref: 10954430 J Cell Sci, 2000
- The findings also suggest that the stress fibers, in which  **actin**  and myosin filaments organize into sarcomere like units with alpha  **actinin**  rich dense bodies analogous to Z lines, are the contractile structures of cultured SMCs that link to the network of vimentin containing intermediate filaments through the dense bodies and dense plaques.  Ref: 11036087 J Histochem Cytochem, 2000
- The high level labeling of alpha SM  **actin**  in the central portion of stress fibers was related to continuous myosin and punctate alpha  **actinin**  distribution, which may represent the maturation of the fibrillar structures.  Ref: 11036087 J Histochem Cytochem, 2000
- Alpha  **actinin**  alpha  **actinin**  2 is a protein which links the NR1 and NR2B subunits of N methyl D aspartate NMDA glutamate receptors to the  **actin**  cytoskeleton.  Ref: 10925145 Brain Res Mol Brain Res, 2000
- The predominant distribution of beta NM  **actin**  in the cell periphery was associated with densely distributed vinculin plaques and disrupted or striated myosin and alpha  **actinin**  aggregates, which may reflect a process of stress fiber assembly during cell spreading and focal adhesion formation.  Ref: 11036087 J Histochem Cytochem, 2000
- CLP36 binds to alpha  **actinin**  1 as shown by coimmunoprecipitation, pull down experiments, yeast 2 hybrid analysis, and blot overlay assays and colocalizes with alpha  **actinin**  1 along endothelial  **actin**  stress fibers.  Ref: 11110697 Blood, 2000
- In the presence of calponin less alpha  **actinin**  was bound to the bundled  **actin**  filaments, and the binding of alpha  **actinin**  was accompanied by a partial decrease in the calponin  **actin**  stoichiometry in the bundles of  **actin**  filaments.  Ref: 10995029 IUBMB Life, 2000
- Human CLP36, a PDZ domain and LIM domain protein, binds to alpha  **actinin**  1 and associates with  **actin**  filaments and stress fibers in activated platelets and endothelial cells.  Ref: 11110697 Blood, 2000

**Parallel relationship** (e.g. studied together, co-existance, homology, etc.)

- Using static light scatter technique, I observed affinities of the order talin > filamin > F  **actin**  > alpha  **actinin**  > talin when bound to vinculin which were lower when integrin was complexed with fibronectin.  Ref: 10799333 Biochem Biophys Res Commun, 2000
- These results suggest that PLD2 is reciprocally regulated in sarcolemmal membranes by alpha  **actinin**  and ARF1 and accordingly that a major role for PLD2 in cardiac function may involve reorganization of the  **actin**  cytoskeleton.  Ref: 10801846 J Biol Chem, 2000
- We have applied fluorescence ratio imaging to the analysis of an  **actin**  binding protein concentration relative to F  **actin**  in macrophages, in order to explore the role of a novel alpha  **actinin**  isoform,  **actinin**  4, relative to that of the classical isoform,  **actinin**  1.  Ref: 10954430 J Cell Sci, 2000
- At low temperatures, for which the lifetime of binding of alpha  **actinin**  to F  **actin**  is long, F  **actin**  alpha  **actinin**  networks exhibit strong strain hardening at short time scales and soften at long time scales.  Ref: 10954703 J Biol Chem, 2000
- Giardia lamblia rearranges F  **actin**  and alpha  **actinin**  in human colonic and duodenal monolayers and reduces transepithelial electrical resistance.  Ref: 10958459 J Parasitol, 2000
- I examined the binding kinetics between integrin alpha IIb beta 3 and purified focal adhesion proteins, including alpha  **actinin** , filamin, vinculin, talin, and F  **actin** .  Ref: 10799333 Biochem Biophys Res Commun, 2000
- The study further shows that CLP36 binds to alpha  **actinin**  in resting platelets and translocates as a CLP36 alpha  **actinin**  complex to the newly formed  **actin**  cytoskeleton in activated platelets.  Ref: 11110697 Blood, 2000
- The ion channels and alpha  **actinin**  2 co localize at the membrane in HEK cells, where disruption of the  **actin**  cytoskeleton and antisense constructs to alpha  **actinin**  2 modulate the ion and gating current density.  Ref: 10812072 FEBS Lett, 2000
- Calponin had no influence on the binding of alpha  **actinin**  to the single  **actin**  filaments.  Ref: 10995029 IUBMB Life, 2000
- When myofibril was incubated with the enzyme, it optically degraded myosin heavy chain at 55 60 degrees C, while alpha  **actinin**  and  **actin**  were not at all hydrolyzed as detected by immunoblotting.  Ref: 10817913 Comp Biochem Physiol B Biochem Mol Biol, 2000
- For F  **actin**  networks in the absence of alpha  **actinin**  or for F  **actin**  alpha  **actinin**  networks at high temperatures, strain hardening appears only at very short time scales.  Ref: 10954703 J Biol Chem, 2000
- These findings demonstrate the first known direct linkage between extracellular EPEC, through the transmembrane protein Tir, to the host cell  **actin**  cytoskeleton via alpha  **actinin** .  Ref: 10873808 Curr Biol, 2000
- The study suggests that CLP36 may direct alpha  **actinin**  1 to specific  **actin**  structures and at this position might modulate the function of alpha  **actinin**  1.  Ref: 11110697 Blood, 2000
- Exposure to live G. lamblia trophozoites induced localized condensation of F  **actin**  and loss of perijunctional alpha  **actinin**  while G  **actin**  remained unchanged.  Ref: 10958459 J Parasitol, 2000
- The results indicate that G. lamblia induced epithelial injury is associated with F  **actin**  and alpha  **actinin**  rearrangements in the terminal web area via mechanisms independent of extracellular calcium.  Ref: 10958459 J Parasitol, 2000
- In the present study we have defined the index of retardation as the concentration of alpha  **actinin**  needed to stop all filament movement, and we propose that this index is a measure of the isometric force exerted by HMM on  **actin**  filaments.  Ref: 10970781 Biochem J, 2000
- The effects of live Giardia lamblia on cellular G  **actin** , F  **actin** , alpha  **actinin** , and electrical resistance of human intestinal epithelial monolayers were investigated using SCBN and Caco2 cell lines grown on chamber slides or Transwell filter membranes.  Ref: 10958459 J Parasitol, 2000
- Like a few F  **actin**  bundling proteins such as HS1, alpha  **actinin** , and vinculin as well as F  **actin**  cappers such as tensin and chaetoglobosin K CK, the F  **actin**  bundling drug MKT 077 suppresses ras transformation by blocking membrane ruffling.  Ref: 10882332 Cancer JCancer J, 2000
- Here, we describe the strain dependence of a model system consisting of  **actin**  filaments F  **actin** , a major component of the cytoskeleton, and the F  **actin**  cross linking protein alpha  **actinin** , which localizes along contractile stress fibers and at focal adhesions.  Ref: 10954703 J Biol Chem, 2000
- Vinculin, alpha  **actinin** , beta dystroglycan and utrophin were all retained on mdx sarcolemma, indicating that the loss of costameric  **actin**  was not due to generalized membrane instability.  Ref: 10974007 J Cell Biol, 2000
- However, ratio images further demonstrated that  **actinin**  4 concentrations relative to F  **actin**  were higher in peripheral inward curved ruffles and dorsal circular ruffles, presumed precursor forms of macropinosomes, than in straight linear ruffles, while  **actinin**  1 concentrations were uniform among the different types of ruffles.  Ref: 10954430 J Cell Sci, 2000
- This PAR 2 mediated increase in keratinocyte phagocytic capability correlated with an increase in  **actin**  polymerization and \*  **actinin**  reorganization, cell surface morphological changes and increased soluble protease activity.  Ref: 10934047 J Cell Sci, 2000
- All CRP family members characterized so far have been shown to interact with the filamentous  **actin**  cross linker alpha  **actinin** .  Ref: 10926853 Biochem J, 2000
